# Global Eradication of Lymphatic Filariasis: The Value of Chronic Disease Control in Parasite Elimination Programmes

**DOI:** 10.1371/journal.pone.0002936

**Published:** 2008-08-13

**Authors:** Edwin Michael, Mwele N. Malecela, Mihail Zervos, James W. Kazura

**Affiliations:** 1 Department of Infectious Disease Epidemiology, Imperial College London, London, United Kingdom; 2 National Institute for Medical Research, Dar es Salaam, Tanzania; 3 Mathematics Department, London School of Economics and Political Science, London, United Kingdom; 4 Center for Global Health & Diseases, Case Western Reserve University, Cleveland, Ohio, United States of America; Yale University, United States of America

## Abstract

The ultimate goal of the global programme against lymphatic filariasis is eradication through irrevocable cessation of transmission using 4 to 6 years of annual single dose mass drug administration. The costs of eradication, managerial impediments to executing national control programmes, and scientific uncertainty about transmission endpoints, are challenges to the success of this effort, especially in areas of high endemicity where financial resources are limited. We used a combined analysis of empirical community data describing the association between infection and chronic disease prevalence, mathematical modelling, and economic analyses to identify and evaluate the feasibility of setting an infection target level at which the chronic pathology attributable to lymphatic filariasis - lymphoedema of the extremities and hydroceles - becomes negligible in the face of continuing transmission as a first stage option in achieving the elimination of this parasitic disease. The results show that microfilaria prevalences below a threshold of 3.55% at a blood sampling volume of 1 ml could constitute readily achievable and sustainable targets to control lymphatic filarial disease. They also show that as a result of the high marginal cost of curing the last few individuals to achieve elimination, maximal benefits can occur at this threshold. Indeed, a key finding from our coupled economic and epidemiological analysis is that when initial uncertainty regarding eradication occurs and prospects for resolving this uncertainty over time exist, it is economically beneficial to adopt a flexible, sequential, eradication strategy based on controlling chronic disease initially.

## Introduction

Given that the health and economic dividends of the current global campaign to eradicate lymphatic filariasis (LF) by mass drug administration (MDA) could be large [1], [2], a strategy that has as its ultimate goal the sustained interruption of transmission of this mosquito-borne infectious disease represents the optimal policy from a social perspective [3]. This optimality, as for any parasite control programme, can be tempered, however, by the technical challenge of maintaining adequate population coverage with MDA necessary to achieve cessation of transmission within the currently mooted period of 4 to 6 years and the financial burden of continuing intervention programmes that target the last few communities and individuals necessary to meet criteria considered to be indicative of eradication [3]. Moreover, achieving parasite eradication implies the need for sustaining control programmes in order to continue surveillance for residual infections beyond 4 to 6 years [4]. Current scientific understanding of LF eradication is also hampered by imperfect knowledge regarding parasite transmission dynamics and endpoint targets [5].

Recent work has also clarified the economic behaviour of investments in parasite eradication [3], [6], [7]. A key notion regarding the desirability of undertaking parasite eradication from this perspective is that incurring immediate costs is expected to yield high future long-term benefits. An important caveat of these appraisals is that most of this work has assumed the technical feasibility of eradication with little focus given so far to the role of payoff uncertainty on the choice of optimal strategies. These gaps in analysis are particularly significant when considering work carried out in the field of ecological and strategic management, which shows that when longer-term investment outcomes are uncertain, it may be optimal to identify and implement effective strategies over the short term, which can be adapted flexibly to achieve investment goals as information regarding the attainability of desired outcomes improves in time [8], [9]–[11].

These considerations suggest that in certain geographical regions, especially in Sub-Saharan Africa and other areas where LF endemicity is high and budgetary and capacity constraints apply, it may be desirable to adopt flexible strategies that first reduce infection to levels that prevent the occurrence of LF disease manifestations even though a steady state, low level of transmission persists. Although it is not yet known whether such a strategy would be successful, empirical observations from some MDA trials have shown that the prevalence of the major chronic disease manifestations of LF, lymphoedema of the extremities and hydrocele, can be decreased without reducing transmission levels to zero [12], [13]. Here, we combine epidemiological analyses of observed data describing the relationship between the overall prevalence of chronic bancroftian filarial disease and microfilarial (mf) infection with mathematical model predictions of filariasis reinfection and economic analysis of MDA interventions, to estimate and clarify the value of using a threshold infection level below which LF-induced pathology becomes negligible as a first stage option in the successful eradication of filariasis.

## Methods

### (a) Data Sources

Field study data (see Table S1 online) on the association between the prevalence of *Wuchereria bancrofti* mf infection and combined lymphoedema and hydrocele disease rates in filarial endemic communities were extracted from the published literature for each of the major endemic regions of Sub-Saharan Africa, Asia other than India, India, Latin America and the Pacific. Although this yielded data from 94 separate communities, perusal of the data showed that adequate data covering the full range of infection prevalence, especially at lower mf prevalence values, existed only for Sub-Saharan Africa and India. Our analysis in this study was therefore by necessity limited to these data compiled from a total of 76 separate communities from these two regions. Prior to analysis, all mf prevalence values were standardized to reflect sampling of 1 ml blood volumes using a transformation factor of 1.95 and 1.15 respectively for values originally estimated using 20 µl or 100 µl blood volumes. These factors were derived using comparative prevalence data from the parallel application of these different diagnostic methods on the same individuals [14]–[18], and the function, 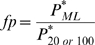, where P^*^_ML_ is the mean mf prevalence obtained using the 1 ml blood filtration method and P^*^_20_ and P^*^_100_ denote the mf prevalences obtained using 20 ul and 100 ul blood volumes [5].

### (b) Statistical Analysis

The association of mf infection with chronic LF disease is described and the existence of a threshold in the relationship was examined via fitting of the following hierarchical logistic dose-response regression models with and without the specification of a threshold parameter to the assembled data [19]–[21]. Model 1 represents the nonthreshold model and is the familiar basic logistic regression model with an intercept relating the prevalence of chronic LF disease to observed community mf prevalence values given by the equation:
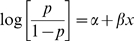
(1)The threshold mf prevalence level (*τ*) at which parasitic infection begins to contribute significantly to the development of chronic filarial disease is then estimated by fitting the corresponding logistic dose-response model which incorporates the threshold parameter as depicted by the equation:
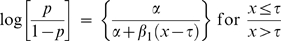
(2)where *p* in both models is the probability of the occurrence of combined lymphoedema and hydrocele in the community, *τ* is the threshold value of the risk factor, *x* (*i.e.* mf prevalence), *a* defines the baseline morbidity prevalence due to non-filarial causes of disease, and *β* and *β*_1_ describe the degree of association between infection and disease with and without the threshold. Model 2 assumes that the risk of chronic disease is constant below the threshold *τ* and increases according to the logistic equation above *τ*. Given prevalence data, we fitted the models assuming binomial errors and obtained parameter estimates for the data by essentially maximizing the log-likelihood function [22]:
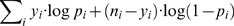
(3)where *y_i_* is the observed disease cases in each community and *p_i_* is the predicted prevalence of disease obtained from equations 1 and 2 respectively, given prevalence of infection, *x*, and *n_i_* is the sample size used for assessing disease from each study. We used the gnlr function in the R software programme, which fits nonlinear regression equations to data for various common one and two parameter error distributions, including the binomial distribution, by minimizing the −log-likelihood via the Newton-Raphson iterative method, to fit both models in this study (see details in [23]). The hypothesis that the threshold model fits the relationship between community mf prevalence and LF disease prevalence better than the nonthreshold model is assessed by comparing the −log likelihood values of each model. The null hypothesis is H_0_: *τ* = 0 and the alternate hypothesis is H_1_: *τ*>0. The test statistic is the likelihood ratio statistic: *LR* = −2(ln *L*(H_0_)−ln *L*(H_1_)), where *L*(H_0_) is the −log likelihood value of the model without a threshold (model 1) and *L*(H_1_) is the −log likelihood value of the model with the threshold level (model 2). The null hypothesis is rejected if the statistic, *LR*, is greater than 3.84 (critical value, α = 0.05). Note that although data from two of the major endemic regions are used, we were able to fit the models to all the available data and hence, effectively estimate the global threshold value for the association between mf prevalence and LF chronic disease for the overall data. Our efforts at fitting the threshold models used in this study to the data when stratified by endemic regions failed to converge, most certainly due to the paucity of such data and especially the restricted ranges of *x*-values (mf prevalences) observed for the available studies at the regional level. As in the case of estimating transmission endpoints [5], this result highlights the need for generating standardized regional data over the full range observed in the field (but especially over the lower mf prevalence range) if these more pertinent regional figures are to be reliably estimated.

### (c) Modelling the impact of MDA interventions

Investigation of the impact of using the estimated mf prevalence threshold for filarial disease as an intervention endpoint in MDA programmes was carried out by simulation using EPIFIL, a deterministic model for LF transmission [5], [24], [25]. Simulations predicting annual changes in overall community mf prevalence following a 5-year intervention programme with either of the two major MDA regimens (*viz.* annual single dose diethylcarbamazine in combination with albendazole (DEC/ALB) or ivermectin in combination with albendazole (IVM/ALB)) with the assumption of a pre-control mf prevalence of 10% were compared for treatment coverages of 65%, 80% and 95%. Drug efficacy values used in the simulations were: DEC/ALB – 55% worm kill, 95% mf cured and 6 months mf suppression; IVM/ALB – 35% worm kill, 99% mf cured and 9 months mf suppression [25].

### (d) Marginal cost-effectiveness analysis

This was performed by combining model predictions of the dynamic impact of an annual IVM/ALB MDA programme given at 80% coverage in curing individuals of mf with the costs of carrying out this MDA programme. The major objective of this analysis was to determine and evaluate the marginal costs of curing additional individuals to achieve parasite elimination and eradication of transmission compared to achieving disease control in a major endemic country. We illustrate the results by basing the analysis on the real life situation in Tanzania, where the endemic pre-control LF infection level has been estimated to be around 11.95% mf prevalence [26]. The analysis assumed that the entire current population of Tanzania above 5 years is at risk and so eligible for treatment (corresponding to 28.79 million as estimated in 2002 [27]). Model predictions of the impact of the annual IVM/ALB MDA given at 80% coverage for a baseline mf prevalence of 11.95% were used in the calculations of the mean number of individuals cured during the MDA programme, with the assumption that LF is eliminated when mean mf prevalence is reduced to <0.5% [5]. Two types of programme costs were estimated and compared. First, the total programme cost accounting for all resources (including drug and programme delivery costs) used in carrying out an annual MDA, which was conservatively assumed to be around $0.70 for treating one individual [28], [29]. Second, government costs, defined as the financial costs of all inputs paid for directly by the Ministry of Health excluding any donations to the programme such as donated drugs. We estimated this cost to be around $0.53 per treated individual using the estimates given in Ramzy and colleagues [29]. The former approach is useful in assessing the allocation of programme resources and their opportunity costs, *i.e.* determining whether these resources could be used more productively elsewhere, whereas the latter cost estimates are helpful to national planners in assessing programme affordability. Numbers of individuals cured and all costs were discounted at 6% [28].

### (e) Modelling the cost of sequential-decision approaches based on chronic disease control

We evaluated the strategic value of implementing a sequential decision approach to LF eradication in which chronic disease control is first achieved followed by additional MDAs to achieve parasite eradication when information regarding the feasibility of eradication improves over time, by undertaking a scenario-based analysis of the comparative costs of a programme aimed from the beginning solely at eradication versus a two-staged strategy based initially on disease control as follows. The analysis was based on EPIFIL predictions of the number of annual MDAs required to meet the endpoints of these scenarios given the baseline endemic mf infection prevalence level of 11.95% estimated for Tanzania and a drug coverage level of 80%.

First, we considered the cost of the base strategy in which we are certain that parasite eradication can be achieved in 10 years. The expected present cost (EPC) of this strategy is given by:
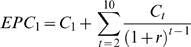
(4)where *C_1_* is the estimated cost of MDA in year 1 (calculated at a per capita cost of $0.70 per person (see above)), *C_t_* denotes the cost of MDA for each of the years 2 to 10, and *r* represents the discount rate (6% as above).

In scenario two, we assume that information regarding the feasibility of eradication becomes clear only some time in the future, say in year 5 following repeated annual MDAs (i.e. at the disease control threshold) when we will either know with a high probability, p = 0.90, that eradication is possible by year 10 in which case we will continue with 5 more annual MDAs to achieve eradication or we will know with a probability 1−p = 0.10 that eradication is not achievable with the current knowledge and/or technology. At this point, we may abandon the goal of eradication and switch to long-term control, i.e implement treatment every 10 years (calculations shown here for at least up to 35 years), thus allowing flexibility to wait for further information to resolve uncertainty to aide making the choice that maximizes the value and minimizes loss from the MDA programme (as a result of reinfection to baseline levels if eradication is not achieved).

In scenario 3, we model a situation similar to scenario 2, but instead of considering that eradication is never possible, we consider that uncertainty in present knowledge and technology is likely to be resolved by years 10 and 15 post initial MDA, when the probability of eradication will occur at p = 0.90, and eradication then given model predictions is expected to be possible with an extra three years of annual MDA following disease control with 5 annual MDAs initially.

The EPC of scenario two is hence given by:
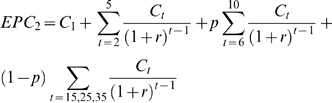
(5)where *C_1_*, *C_t_* and *r* are described as above and *p* denotes the probability that eradication is possible (p = 0.90).

For scenario three, in which disease control is followed by the switch to eradication when information regarding the feasibility of eradication becomes better known either in year 10 or year 15, the EPC for the year 10 case is given by:
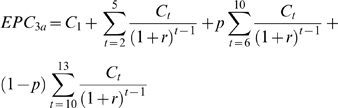
(6)and for the year 15 case by:
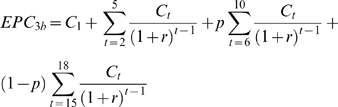
(7)with variables as described before.

## Results

**Figure 1:**
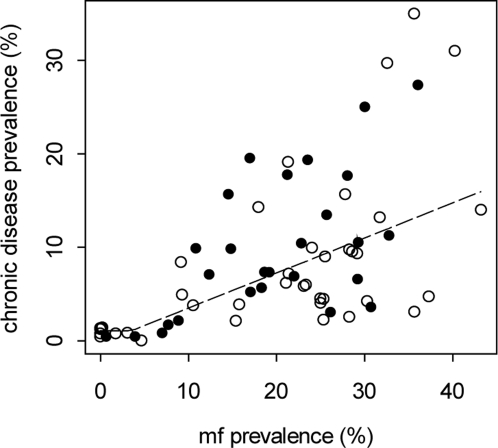
The association between prevalence of *Wuchereria bancrofti* microfilarial infection and prevalence of combined lymphoedema and hydrocele disease in filarial endemic communities from Sub-Saharan Africa (○) and India (•). Field study references (n = 76) are given in Table S1 online. All mf prevalence values have been standardized to reflect sampling of 1 ml blood volumes (see text). The curve shows the best-fit logistic regression-based dose-response model incorporating a threshold parameter (equation 2) for the overall data with estimated values for *a* and τ of 1.07% (95% confidence limits (CLs): 0.54 to 1.60%) and 3.55% (95% CLs: 2.35 to 4.75%), respectively. Details of goodness of fit of the model are given in Supplemetary figure 1 online.

Figure 1 depicts the data collated from all relevant published studies available from Sub-Saharan Africa and India on the association between the community prevalence of LF infection and the corresponding prevalence of chronic disease (*n* = 76). Despite the presence of some between-region geographic variation, the scatterplot depicted in the figure shows that there is a global occurrence of an overall positive but non-linear association between chronic disease attributed to LF and mf infection, with disease apparently constant up to a threshold mean mf prevalence level and then increasing positively. Such a disease-infection pattern, shown here for the first time for LF, suggests the operation of an infection dose response function whereby in communities with low mf prevalence the observed disease may be due to causes other than filariasis while disease attributable to filarial infection develops only above a specific mf threshold [22]. Table 1 shows the results of the statistical fits of models 1 and 2 to the data, and supports the visual impression from figure 1 of the existence of an “average” mf threshold in the development of LF chronic disease in the present data. The curve in the figure portrays the predictions of the logistic regression-based dose response model incorporating a threshold (model 2), and indicates that the pattern illustrated by the data for the occurrence of a threshold dependent dose-response in the association between LF infection and disease is adequately described by this model (see Text S1 and Figure S1 online for details of goodness of fit of the model). This result indicates that a threshold mf prevalence value that could be used as a target for suppressing formation of filarial disease may lie in the region of 3.55% (95% confidence limits: 2.35–4.75) at the 1 ml blood sampling volume scale, with the caveat that there could be regional differences in the value of this threshold among endemic communities based on the peculiar features of local mosquito-vector parasite interactions. The results also indicate that on average up to 1.07% of the observed lymphoedema and hydrocele cases may be non-filarial in aetiology [30].

**Table 1:** Parameters and negative log-likelihoods for the logistic dose-response models with and without a threshold fitted (see text) to the mf – LF chronic disease prevalence data (*n* = 76).

	Value of:			−log likelihood	*LR* 1
Model	*α*	*β*	*τ*		
1	1.02 (0.096)	0.27 (0.005)	-	2720.635	
2	1.07 (0.530)	0.34 (0.008)	3.55 (1.196)	2230.311	15.817^2^

Figures in parentheses denote the standard errors estimated for each parameter.

1 Likelihood ratio statistic (see text).

2 *p*<0.0001.

**Figure 2:**
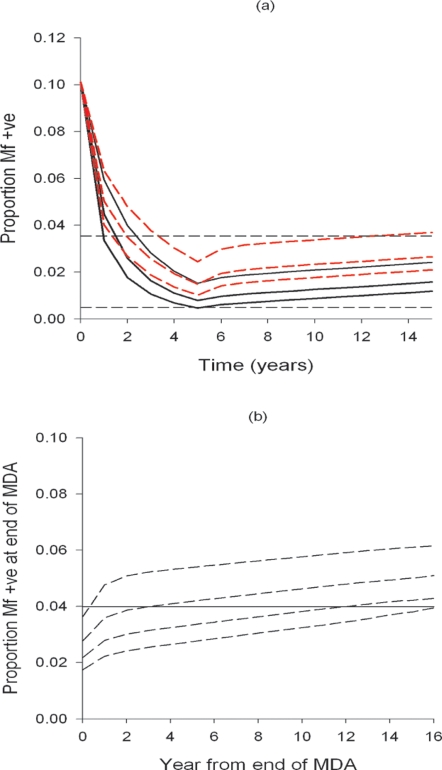
(A) Simulation results showing annual changes in overall community mf prevalence (scaled to 1 ml blood sampling volume) predicted by a deterministic model for filariasis transmission following a 5-year annual intervention programme with either the DEC/ALB (solid lines) or IVM/ALB (dashed lines) drug regimen. Initial community mf prevalence was set at 10%, and for each regimen predictions are shown for treatment coverages of 65%, 80% and 95% (portrayed by curves going from top to bottom respectively for each regimen). Upper horizontal dashed line shows the disease control mf threshold of 3.55%, while the lower line depicts the parasite elimination threshold of 0.5% mf prevalence. All figures are given at the scale of 1 ml blood sampling volume. Drug efficacy values are as given the text. (B) Numerical projections (dashed curves) of changes in LF infection prevalence (Mf % at 1 ml blood sampling scale) following different levels of reduction from initial equilibrium infection prevalence induced by annual MDA, as predicted by the EPIFIL model. Note that the greater the reduction of initial prevalence to below the mean disease threshold of 3.55% mf prevalence (solid horizontal line), the longer it will take to rebound to this threshold. For example, a reduction to a mf prevalence of 2.175% following annual MDA (second dashed curve from the bottom) will take up to 12 years to rebound to the disease incidence threshold.

Figure 2A shows how predictions of a mathematical model of filariasis transmission [24], [25], [31] allow an examination of the usefulness of implementing a 5-year MDA strategy with DEC/ALB or IVM/ALB that has as its target either parasite elimination and eradication (set to be around 0.5% mf prevalence here [5]) or disease control (3.55% mf prevalence). The simulations in the figure are all based on a moderately high overall community pre-control mf prevalence of 10% (at the scale of 1 ml blood sampling volume), and for each treatment regimen are illustrated for coverage values of 65%, 80% and 95% (portrayed by curves going from bottom to top respectively for each drug regimen). The results indicate that while neither MDA regimen achieved the parasite elimination target for all the three treatment coverages considered, both achieved the disease control target of 3.55% mf prevalence before 5 years. This was true even in the case of the less effective IVM/ALB regimen given at the lowest MDA coverage of 65%. An additional feature of the results is that following the end of the treatment programme, the rates of rebound of infection (given that transmission has not been interrupted) are also predicted to be slow. For the IVM/ALB regimen with only 65% population coverage, infection levels reduced to ∼2.35% are predicted to remain under the disease control threshold for at least 7 years after cessation of the 5-year intervention programme. At higher coverages, and for the more effective DEC/ALB regimen, infection levels remained below the threshold level for more than 10 years (Fig. 2A).

This effect of the achieved post-control mf prevalence following MDA on the rate of return of infection prevalence to pre-control equilibrium values is more clearly shown in Figure 2B, and indicates how this relationship can be used to guide the setting of the mf prevalence target for long-term disease control. Thus, the numerical projections in Figure 2B indicate that reducing mf prevalence to below 2.35% (i.e. below the lower 95% CL value of the disease control threshold (Table 1)) may maintain infection levels below the disease incidence threshold of 3.55% mf prevalence for up to as long as ∼10 to 12 years before repeat MDAs will be required to depress infection again to sustain the control of disease. This dynamical epidemiological result clearly makes disease control both a feasible and sustainable option in endemic communities.

Table 2 summarizes and compares the predicted average and marginal costs of the annual IVM/ALB MDA programme (80% coverage) in curing individuals of mf to achieve LF elimination in Tanzania. The results show that for both types of cost estimations (the total economic and the financial costs of programme delivery only by the Ministry of Health), the average cost per individual cured of mf to achieve disease control will be moderately low whereas the average and marginal cost of curing additional individuals at each subsequent MDA round until transmission elimination is achieved will increase steeply. This is because, as the number of MDA rounds increase (an additional 5 years of MDA is predicted to be required to achieve transmission elimination as compared to 5 years of MDA in total to achieve long-term disease control, ie., by achieving mf prevalence reduction to 2.35% following annual MDA (see above)), a progressively decreasing proportion of individuals are cured at the same fixed outlay for implementing the annual mass treatment programme. For example, only an extra 0.32 million individuals are cured of mf over the additional 5 MDAs to meet the target of transmission elimination as compared to the 2.5 million cured over 5 years to achieve long-term disease control (Table 2). Because the number of MDA rounds required to achieve disease control is smaller than that required to achieve transmission elimination (indeed, in general this will take half the time required for parasite elimination (results not shown)), the marginal cost of achieving disease control will also be considerably smaller compared to that for achieving parasite elimination. The analysis presented here used a very conservative cost of $0.70 in the case of the total economic cost approach and $0.53 in the case of the Government cost method to treat one individual [28], [29], [32], [33]; increasing this cost will only further amplify the present findings.

**Table 2:** Predicted average and marginal costs of an annual IVM/ALB MDA programme to control disease or eliminate LF transmission in the Republic of Tanzania.

Programme	Cost (US$)^3^	No. of individuals cured of mf	Ratio of total cost to no. of individuals cured of mf (US$ per individual)^3^	Ratio of Govt cost to no. of individuals cured of mf (US$ per individual)^4^
1. Disease control (5 years of annual MDA)^1^	89,949,942	2,467,830	36.45	27.60
2. Transmission Elimination (10 years of annual MDA)^2^	157,165,771	2,790,395	56.32	42.65
3. Increment (of Programme 2 over Programme 1)^5^	67,215,829	322,565	208.38	157.77

1 Represents a programme targeted at disease control assumed to occur when the mf prevalence is reduced long-term (for at least 10 years) to below 3.99% (figure 2a). For a baseline prevalence of 11.95% mf prevalence, it is predicted to take up to 5years of MDA with IVM/ALB to reduce infection prevalence to 2.3% to achieve this objective at 80% coverage.

2 Represents a programme to eliminate parasite transmission assumed to occur when the mf prevalence is reduced to just below 0.5%. For a baseline prevalence of 11.95% mf prevalence, it is predicted to take up to 10 years of MDA with IVM/ALB to achieve this target at 80% coverage.

3 The full economic cost of the programme, including the cost of donated drugs and programme delivery costs borne by the Government estimated at $0.70 for treating each individual.

4 Excludes the cost of donated drugs. Estimate of drug costs to total programme cost obtained from ref (24.2%) is used in calculating only the additional Government cost involved in delivering the MDA programme. Effectively, this decreases the per capita cost of treating an individual from $0.70 per individual for the total cost estimation to $0.53 per individual for the Government costs only estimation.

5 Addition individuals cured of mf and the total and average marginal costs of curing these individuals to achieve parasite transmission elimination from a programme targeted at disease control.

**Figure 3:**
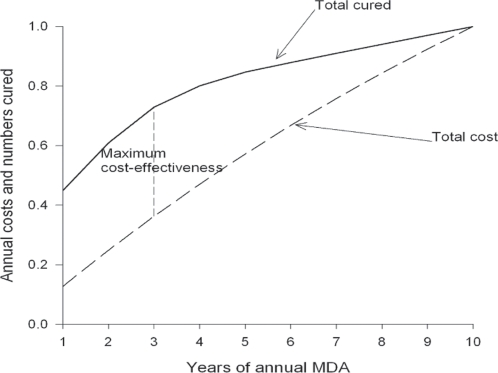
Marginal effectiveness (numbers of individuals cured of mf) and costs of annual mass IVM/ALB chemotherapy against LF in Tanzania and the determination of the optimal level of parasite control. Simulations of costs and effectiveness are based on the cost and demographic details given in table 2 and the impact of annual IVM/ALB mass treatment (at 80% coverage) on the prevalence of LF infection predicted by EPIFIL assuming the baseline Mf prevalence of 11.95% (at 1 ml blood sampling volume) in Tanzania. Annual costs and effectiveness are both expressed as a fraction of their total discounted figures following 10 years of intervention (table 2). The results show that the maximum number of individuals cured of mf per unit cost occurs at 85% parasite control following 3 years of annual MDA closer to achieving the long-term disease control target of 2.35% (see text) than the target of 100% parasite control or elimination.

Indeed, in cost-effectiveness terms, the declining number of additional individuals cured as annual MDA proceeds compared to the increasing marginal cost of curing these individuals from the additional cycles of MDA suggests that maximal benefits may occur at less than 100% parasite control or parasite elimination. This is highlighted in Figure 3, which graphically compares the expected effectiveness and costs of the annual MDA programme to eliminate LF in Tanzania. Figure 3 shows that the maximal cost-effectiveness of the programme could occur at 85% parasite control following 3 annual MDAs. This is closer to the predicted 5 cycles of annual MDA required to achieve long-term disease control for this country (Table 2) in contrast to the 10 annual cycles needed to achieve parasite elimination or 100% parasite control, and further supports the soundness of a disease control option as a first phase intermediate objective in efforts to eradicate LF.

This incremental role of controlling disease first in LF eradication is reinforced by the estimates of the expected present costs (EPCs) of LF eradication strategies for the Republic of Tanzania with and without a flexible sequential-decision option based on exercising disease control option, shown in Table 3. The number of years needed to achieve LF disease control or/and eradication were based on EPIFIL model predictions as described in **Methods**, while similar treatment costs and discount rates as utilized in the marginal cost-effectiveness analysis were used in the analysis for comparative purposes. The results depict that the EPCs of strategies that contain the flexibility of switching between disease control and eradication are lower than that of an eradication strategy in which no flexibility to either switch to disease control if eradication is not feasible or to embark upon eradication after disease control when eradication becomes favourable exists. Since the EPCs of the flexible strategies are lower than that of the inflexible eradication strategy, it is clear that such strategies should be preferable highlighting their optimality when uncertainty regarding eradication occurs. Indeed, the results show that switching to long-term disease control from year 5 (MDAs given every 10 years following an initial 5 annual MDAs to achieve disease control: Strategy 2) can be the cheapest intervention option for LF if eradication can never be achieved (Table 3), while the lower EPCs of Strategies 3a & b compared to Strategy 1 highlight the costs which can be saved by waiting for better information regarding the feasibility of eradication before switching to eradication from disease control. The value of including such flexibilities in the LF eradication programme for the republic of Tanzania is given by option value figures depicted in Table 3, and indicates that including flexible decision-making based initially on disease control can yield savings of between US$ 3.3 to 5.0 million depending on the feasibility of eradication (from never (Strategy 2) to waiting to switch to eradication at different times when knowledge and technology improves (Strategies 3a & 3b)).

**Table 3:** Expected Present Costs of LF eradication strategies for the Republic of Tanzania with and without sequential-decision making flexibility based on exercising chronic disease control options.

Strategy^1^	Design	EPC^2^	Option Value^3^
S1	*Inflexible*: 10 year annual MDA	157,165,771	
S2	*Flexible*: Switch to long-term disease control after 5 years of annual MDA when it becomes evident eradication is not possible	152,110,572	5,055,199
S3_a_	*Flexible*: Switch to eradication with 3 extra annual MDAs from year 10 following disease control with 5 initial annual MDAs	153,822,682	3,343,089
S3_b_	*Flexible*: Switch to eradication with 3 extra annual MDAs from year 15 following disease control with 5 initial annual MDAs	152,968,795	4,196,976

1 Denotes the three different scenarios described in detail in the text.

2 Expected Present Cost of each strategy in US$.

3 The difference between the EPCs of flexible strategies 2 to 3 over strategy 1 represents the value (costs saved here) of retaining the option to switch between eradication and disease control depending on the state of eradication feasibility.

**Figure 4:**
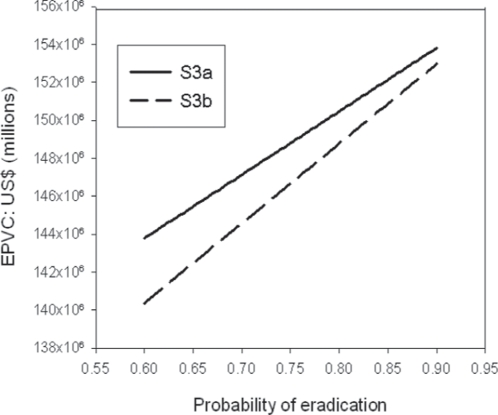
The relationship between Expected Present Costs (EPCs) of implementing Strategies 3a (S3a) and 3b (S3b) (see text) and the probability of LF eradication for the Republic of Tanzania. The lines show that as the uncertainty regarding the feasibility of eradication increases (probability of eradication becomes lower) the expected costs of these flexible strategies decline dramatically.

Figure 4 shows the relationship between the probability of eradication (the lower this probability the higher the uncertainty regarding the feasibility of eradication) and the EPC values of Strategies 3a and 3b. The results show that as uncertainly regarding eradication increases the EPCs of these strategies will decline dramatically, indicating the important result that inclusion of flexibility using chronic disease control should be even more preferable when uncertainty regarding eradicability is high. For example, when uncertainty regarding eradication is even moderately high, say at the probability of eradication of only p = 0.70, the costs saved (or option value of) employing Strategy 3a over the inflexible Strategy 1 can be as high as US$10.0 million and for Strategy 3b even higher at US$12.0 million. Note that because of the effects of discounting, Strategy 3b will always be better value than Strategy 3a as the extra costs related to switching to eradication occurs 5 years later compared to the case with the latter strategy (Table 3).

## Discussion

This study set out to use a combined epidemiological and economic approach in order to evaluate the possibility of estimating and setting an infection threshold that achieves control of LF-related disease as a rational and feasible first stage target for LF eradication programmes. Our analyses show that not only may such a threshold exist but also that this endpoint is likely to be sustainable owing to the slow rebound of infection following MDA (principally as a result of the long life span of the adult parasite [24]). The results also reveal that a third epidemiological factor which may make an intervention strategy aimed at controlling LF morbidity as an intermediate step on the path to parasite eradication attractive to policy makers and health care planners in developing countries – namely that disease control thresholds can generally be achieved in half the time required for accomplishing transmission elimination. This is shown to be the case even if the long-term disease control mf prevalence target is set to be lower (eg, 2.35%) than the infection threshold (3.55%) at which disease develops in communities. From the perspective of national health agencies responsible for implementing and sustaining MDA programmes, this finding highlights the feasibility of achieving disease control readily compared to transmission elimination, especially when model predictions and available data [13], [34]–[37] suggest that even when high population coverage is achieved the duration of annual MDA necessary to stop transmission can be expected to extend beyond 7 years in the many endemic areas where the baseline mf prevalence is equal to or greater than 10% [5], [25].

Note that our estimate of the disease control threshold was technically made possible only via synthetic epidemiological analysis based on using all the available published data from only two of the major LF endemic regions. Given the geographic variation in the data used to construct Figure 1 and lack of adequate information within and across many other LF endemic areas, this estimated disease threshold could in reality vary between regions. However, as noted above, the paucity of regional data describing the mf and disease prevalence relationship particularly at the lower range of the two variables currently precludes the estimation of pertinent region-specific figures and determination of whether the mf threshold estimated here is valid for all endemic areas that have or will soon participate in national MDA programmes. This conclusion, as highlighted also for the estimation of transmission endpoints in LF [5], signifies the need for collecting more reliable standardized data spanning the natural range of prevalences observed in the field at the regional level. In the meanwhile, given that data from Sub-Saharan Africa were used in the estimation of the disease threshold value here, we suggest that the present results for the Republic of Tanzania are unlikely to be severely influenced by this issue.

The economic analyses carried out in this study have yielded two major insights regarding the choice of optimal eradication strategies for LF hitherto not fully recognized. First, marginal cost-effectiveness analysis has demonstrated that when epidemiological results are combined with economic rationalities, disease control as a first stage target is likely to be attractive to policy makers as a result of the far higher marginal cost that arises from having to cure additional individuals in order to stop parasite transmission completely compared to controlling disease. Coupled with relatively low cost social efforts that emphasize local hygiene to prevent and treat forms of chronic morbidity such as lymphoedema of the extremity [38], this important outcome suggests that the decision to undertake parasite eradication must take explicit account of the opportunity cost of eradication, especially when budgetary and capacity constraints exist within the health systems expected to implement and sustain mass intervention programmes. Indeed, our analysis shows that the relationship between the marginal social benefits and costs of annual LF MDA is such that the optimal level of parasite control may be closer to that achieved by disease control compared to parasite elimination. This result is tempered by the technical feasibility of achieving parasite elimination [3], but it does indicate that given the slow expected rebound of infection when parasite transmission is reduced but not eliminated, human and financial resources that would otherwise be expended for continuation of MDA and infection surveillance during the period of low reinfection could be spent more beneficially on economic and social development of target populations (better sanitation, improved health and educational facilities, increased income-generating options). In purely economic terms, these findings pertaining to both the feasibility and opportunity cost of achieving parasite elimination underscore the well-known principle that opting for first-best efforts, such as eradication or elimination, is unlikely to be attainable in a second-best world [39]–[41]. Instead, they suggest that Pareto optimality under these circumstances may be better achieved by opting for second-best strategies, such as disease control, ie., optimal policy must crucially account for the feasibility of implementing proposed strategies [40]–[42].

Although the results of the marginal cost-effectiveness analysis carried out here has shed light on issues underlying the economic optimality of undertaking disease control versus eradication, particularly that social welfare may be maximized at levels of parasite control below eradication levels, it is restricted in its scope as it does not address the continuous nature of investment that would be required to maintain disease control (which clearly makes eradication optimal if technically feasible [43], [44]) and the issue of uncertainty in the possibility of achieving eradication. The latter is an overriding issue for the development of optimal LF intervention strategies given the current imperfect scientific knowledge regarding transmission endpoint values, the duration and intensity of treatments required to achieve parasite elimination under different endemic conditions, and the potential for the emergence of drug resistance in treated worm populations [5], [25], [45], [46], and problems associated with organizational capacities and finances in many endemic countries that are likely to affect the adequate implementation of long-term interventions.

We have attempted to address these issues via expected cost comparisons of an intervention aiming solely at eradication with those combining disease control with wait options to switch to eradication depending on when knowledge and/or technology for achieving eradication becomes more certain. Our primary finding is that when longer-term uncertainty about programme payoffs exists, there is value in developing and implementing flexible, dynamic approaches that focus on identifying and implementing optimal shorter-term strategies (such as disease control considered here) which can be adjusted (switched to eradication or even long-term disease control) according to future scientific progress [9]–[11]. Our analysis illustrates that this is basically because any long-term decision taken now under uncertainty has an opportunity cost in that it eliminates the option of waiting for further information and hence the possibility of making better loss minimizing decisions later. We have shown that this option value for flexible sequential-decision making under eradication uncertainty can be large depending on the nature and timing of flexibility and degree of uncertainty (Table 3); indeed we calculate that for moderate uncertainty (probability of eradication = 0.70), implementing flexible LF eradication strategies based on chronic disease control initially can yield cost savings (or option values) of up to US$12.0 million over the cost of implementing the alternate inflexible strategy focused solely on eradication currently being recommended for the Republic of Tanzania (Fig. 4).

Analyses carried out in this study and the considerations above suggest that although the aim of parasite eradication by the current global initiative against LF is sound, laudable and likely achievable in areas of low to moderate endemicity, a strategy focused primarily or solely on eradication in every location, especially those where the baseline mf prevalence is high, could be sub-optimal. Rather, we have shown that under those circumstances and especially when there is initial uncertainty and there exists a possibility that knowledge and/or technology for effectively achieving eradication will improve with time, it is more desirable and cost-efficient to achieve disease control at the first instance. This can then be followed by a decision to intensify eradication attempts once baseline mf prevalences are reduced below the disease control threshold of 3.55%. In this respect, it is important to acknowledge how the prospects of harnessing existing and new knowledge regarding chronic LF morbidity control and transmission interruption, particularly the demonstrated value of local hygiene in treating lymphoedema [38], [47], [48], confirmation that MDA prevents and possibly reverses pre-existing LF-related lymphedoema and/or hydrocele [13], and a more rapid achievement of transmission cessation by adding vector control such as insecticide treated bed nets to MDA [49], [50], into the global strategy are likely to improve the feasibility of effective and sustainable parasite eradication. It is also instructive to note that morbidity due to blindness was achieved much more readily than transmission interruption in the case of the intervention programme against the closely related filarial disease, onchocerciasis, in both Africa and Latin America [51], [52]. Finally, it is important to recognize that parasite management programmes are embedded within and affected by real-world social-ecological systems [53], [54]. Thus, implementing a sequential approach will support effective management of these programmes by allowing the incremental achievement of success by health agencies with its attendant boosting of organizational morale, accountability, learning and competency [55], [56].

We conclude that these results indicate that current debates about disease control and eradication must include and take careful account of these factors so that richer and more comprehensive evaluative frameworks than simple epidemiological and investment appraisals can be developed and applied to achieve the control or elimination of human infectious diseases, particularly those occurring in the developing world.

## Supporting Information

Table S1 Details of data and studies used in the analysis shown in Figure 1.
(0.17 MB DOC)

Text S1 Adequacy of fit of the logistic dose-response regression model with a threshold
(0.03 MB DOC)

Figure S1 Logit proportions ( = log odds) of chronic LF disease against individual study (open circles) and pentiles (closed circles) of mf prevalence (%) values. The figures shown on the graph represent the chi-square statistic and p - values obtained by applying the chi-square test described in the text for data grouped into pentiles of mf prevalence.
(6.73 MB DOC)
