# Supplementary material for: Global Eradication of Lymphatic Filariasis: The Value of Chronic Disease Control in Parasite Elimination Programmes
Source: PLoS One. 2008 Aug 13;3(8):e2936. doi: 10.1371/journal.pone.0002936 (PMC2490717; doi:10.1371/journal.pone.0002936)
Supplement: Table S1 — Details of data and studies used in the analysis shown in Figure 1. (0.17 MB DOC) [file pone.0002936.s001.doc]

| Study no. | Reference  No. | Region | Sample  size | Blood  Volume  (μl)^1^ | Overall  Mf prevalence (%)^2^ | Chronic  Disease  Prevalence  ( %)^3^ |
| --- | --- | --- | --- | --- | --- | --- |
| 1 | 1 | Africa | 787 | 100 | 24.98 | 4.32 |
| 2 | 1 | Africa | 1007 | 100 | 25.35 | 4.27 |
| 3 | 2 | Africa | 1129 | 100 | 15.79 | 3.72 |
| 4 | 3 | Africa | 469 | 100 | 0.00 | 0.43 |
| 5 | 3 | Africa | 411 | 100 | 0.00 | 0.97 |
| 6 | 3 | Africa | 524 | 100 | 10.53 | 3.63 |
| 7 | 3 | Africa | 575 | 100 | 21.40 | 6.68 |
| 8 | 3 | Africa | 418 | 100 | 23.11 | 5.50 |
| 9 | 3 | Africa | 528 | 100 | 29.19 | 8.52 |
| 10 | 3 | Africa | 267 | 100 | 0.00 | 0.75 |
| 11 | 3 | Africa | 305 | 100 | 0.00 | 1.31 |
| 12 | 3 | Africa | 262 | 100 | 1.76 | 0.76 |
| 13 | 4 | Africa | 848 | 100 | 28.61 | 8.71 |
| 14 | 4 | Africa | 825 | 100 | 3.07 | 0.81 |
| 15 | 5 | Africa | 489 | 100 | 35.65 | 25.91 |
| 16 | 5 | Africa | 335 | 100 | 40.25 | 23.67 |
| 17 | 6 | Africa | 1603 | 100 | 37.30 | 4.50 |
| 18 | 7 | Africa | 296 | 100 | 30.30 | 4.05 |
| 19 | 8 | Africa | 367 | 100 | 32.59 | 22.89 |
| 20 | 8 | Africa | 546 | 100 | 27.80 | 13.53 |
| 21 | 8 | Africa | 642 | 100 | 21.32 | 16.04 |
| 22 | 8 | Africa | 448 | 100 | 17.97 | 12.50 |
| 23 | 9 | Africa | 1947 | 100 | 24.04 | 9.04 |
| 24 | 10,11 | Africa | 582 | 20 | 15.40 | 2.07 |
| 25 | 10,11 | Africa | 125 | 20 | 4.68 | 0.00 |
| 26 | 10,11 | Africa | 925 | 20 | 28.27 | 8.87 |
| 27 | 10,11 | Africa | 92 | 20 | 25.35 | 2.10 |
| 28 | 12 | Africa | 1418 | 20 | 25.03 | 3.88 |
| 29 | 13 | Africa | 2552 | 20 | 21.09 | 5.80 |
| 30 | 14 | Africa | 1307 | 20 | 35.66 | 2.98 |
| 31 | 14 | Africa | 607 | 20 | 28.27 | 2.47 |
| 32 | 15 | Africa | 180 | 50 | 25.53 | 8.25 |
| 33 | 15 | Africa | 274 | 50 | 43.24 | 12.28 |
| 34 | 15 | Africa | 370 | 50 | 31.74 | 11.64 |
| 35 | 16 | Africa | 1275 | 60 | 23.36 | 5.65 |
| 36 | 16 | Africa | 614 | 60 | 0.18 | 1.30 |
| 37 | 16 | Africa | 471 | 60 | 9.28 | 4.67 |
| 38 | 16 | Africa | 452 | 60 | 9.16 | 8.85 |
| 39 | 16 | Africa | 505 | 60 | 0.23 | 1.38 |
| 40 | 17 | Asia | 353 | 1000 | 11.33 | 4.53 |
| 41 | 18,19 | Asia | 460 | 1000 | 16.09 | 5.39 |
| 42 | 20,21 | Asia | 1708 | 20 | 33.22 | 6.32 |
| 43 | 22 | Asia | 90 | 20 | 28.17 | 4.44 |
| 44 | 22 | Asia | 73 | 20 | 29.38 | 4.11 |
| 45 | 22 | Asia | 122 | 20 | 46.35 | 5.73 |
| 46 | 23 | Asia | 536 | 20 | 8.78 | 2.65 |
| 47 | 24,25 | Asia | 1443 | 20 | 31.49 | 21.05 |
| 48 | 26 | Asia | 590 | 40 | 25.73 | 1.02 |
| 49 | 27 | Asia | 1549 | 40 | 15.07 | 8.26 |
| 50 | 28 | India | 9624 | 15-20 | 32.78 | 10.11 |
| 51 | 29,30 | India | 34615 | 20 | 12.36 | 6.59 |
| 52 | 31 | India | 11300 | 20 | 29.22 | 6.17 |
| 53 | 32 | India | 3098 | 20 | 17.06 | 4.94 |
| 54 | 33 | India | 1555 | 20 | 28.0 | 15.00 |
| 55 | 34 | India | 8386 | 20 | 3.95 | 0.42 |
| 56 | 35 | India | 9012 | 20 | 14.54 | 13.54 |
| 57 | 36 | India | 11212 | 20 | 14.84 | 8.94 |
| 58 | 36 | India | 1838 | 20 | 19.20 | 6.80 |
| 59 | 37 | India | 2716 | 20 | 36.08 | 21.47 |
| 60 | 38 | India | 22334 | 20 | 25.70 | 11.85 |
| 61 | 39 | India | 2999 | 20 | 18.66 | 6.84 |
| 62 | 40 | India | 17717 | 20 | 10.85 | 8.97 |
| 63 | 41 | India | 2658 | 20 | 23.55 | 16.19 |
| 64 | 42 | India | 7976 | 20 | 22.83 | 9.42 |
| 65 | 43 | India | 3486 | 20 | 30.04 | 19.99 |
| 66 | 44 | India | 7402 | 20 | 29.29 | 9.51 |
| 67 | 45 | India | 4664 | 20 | 21.26 | 15.07 |
| 68 | 45 | India | 4659 | 20 | 18.33 | 5.34 |
| 69 | 46 | India | 8581 | 20 | 22.02 | 6.43 |
| 70 | 47 | India | 522 | 20 | 7.02 | 0.8 |
| 71 | 47 | India | 203 | 20 | 30.74 | 2.52 |
| 72 | 47 | India | 291 | 20 | 26.13 | 2.95 |
| 73 | 47 | India | 2384 | 20 | 0.65 | 0.17 |
| 74 | 47 | India | 3163 | 20 | 8.88 | 2.09 |
| 75 | 48 | India | 5883 | 20 | 7.72 | 1.67 |
| 76 | 49,50,51 | India | 582 | 40 | 17.00 | 16.33 |

^1^ Figures denotes the blood sampling volume used to estimate Mf prevalence

^2^ Mf prevalence scaled to 1ml blood sampling volume from original volume using the factors given in the text.

^3^ Includes both hydrocele and lymphoedema

Reference List for studies used pertaining to Reference no. in Table S1

1. Wijers DJ, Kinyanjui H (1977) Bancroftian filariasis in Kenya II. Clinical and parasitological investigations in Mambrui, a small coastal town, and Jaribuni, a rural area more inland (Coast Province). Ann Trop Med Parasitol 71: 333-345.

2. Estambale BB, Simonsen PE, Knight R, Bwayo JJ (1994) Bancroftian filariasis in Kwale District of Kenya. I. Clinical and parasitological survey in an endemic community. Ann Trop Med Parasitol 88: 145-151.

3. Dunyo SK, Appawu M, Nkrumah FK, Baffoe-Wilmot A, Pedersen EM, et al. (1996) Lymphatic filariasis on the coast of Ghana. Trans R Soc Trop Med Hyg 90: 634-638.

4. Michael E, Simonsen PE, Malecela M, Jaoko WG, Pedersen EM, et al. (2001) Transmission intensity and the immunoepidemiology of bancroftian filariasis in East Africa. Parasite Immunol 23: 373-388.

5. Meyrowitsch DW, Simonsen PE, Makunde WH (1995) Bancroftian filariasis: analysis of infection and disease in five endemic communities of north-eastern Tanzania. Ann Trop Med Parasitol 89: 653-663.

6. Gyapong JO, Magnussen P, Binka FN (1994) Parasitological and clinical aspects of bancroftian filariasis in Kassena-Nankana District, upper east region, Ghana. Trans R Soc Trop Med Hyg 88: 555-557.

7. Dzodzomenyo M, Dunyo SK, Ahorlu CK, Coker WZ, Appawu MA, et al. (1999) Bancroftian filariasis in an irrigation project community in southern Ghana. Trop Med Int Health 4: 13-18.

8. McMahon JE, Magayauka SA, Kolstrup N, Mosha FW, Bushrod FM, et al. (1981) Studies on the transmission and prevalence of Bancroftian filariasis in four coastal villages of Tanzania. Ann Trop Med Parasitol 75: 415-431.

9. Matola YG (1985) Prospects of human malaria and Bancroftian filariasis infections in the Lower Rufiji Basin, Tanzania II. Bancroftian filariasis. Trop Geogr Med 37: 108-113.

10. Brinkmann UK (1977) Epidemiological investigations of Bancroftian filariasis in the coastal zone Liberia. Tropenmed Parasitol 28: 71-76.

11. Kuhlow F, Zielke E (1976) Distribution and prevalence of Wuchereria bancrofti in various parts of Liberia. Tropenmed Parasitol 27: 93-100.

12. Udonsi JK (1988) Bancroftian filariasis in the Igwun Basin, Nigeria. An epidemiological, parasitological, and clinical study in relation to the transmission dynamics. Acta Trop 45: 171-179.

13. Akogun OB (1991) Filariasis in Gongola State Nigeria. I: Clinical and parasitological studies in Mutum-Biyu district. J Hyg Epidemiol Microbiol Immunol 35: 383-393.

14. Brengues J (1975) La filariose de Bancroft en Afrique de l'Ouest. Memoires d'Orstom 79: 1-299.

15. Simonsen PE, Meyrowitsch DW, Makunde WH, Magnussen P (1995) Bancroftian filariasis: the pattern of microfilaraemia and clinical manifestations in three endemic communities of Northeastern Tanzania. Acta Trop 60: 179-187.

16. Brengues J, Subra R, Bouchite B (1969) Etude parasitologique, clinique et entomologique sur la filariose de Bancroft dans le sud du Dahomey et du Togo. Cah ORSTOM ser Entomol Med Parasitol 7: 279-305.

17. Itoh M, Weerasooriya MV, Gunawardena NK, Mudalige MP, Samarawickrema WA, et al. (1999) Wuchereria bancrofti antigenaemia in Sri Lanka. Trop Med Int Health 4: 207-210.

18. Grove DI, Valeza FS, Cabrera BD (1978) Bancroftian filariasis in a Philippine village: clinical, parasitological, immunological, and social aspects. Bull World Health Organ 56: 975-984.

19. Valeza FS, Grove DI (1979) Bancroftian filariasis in a Philippine village: entomological findings. Southeast Asian J Trop Med Public Health 10: 51-61.

20. Fan PC, Wang YC, Liu JC, Hsu J (1974) Filariasis on Kinmen (Quemoy) Islands, Republic of China I. Parasitological investigation. Southeast Asian J Trop Med Public Health 5: 211-222.

21. Fan PC, Wang YC, Liu JC, Hsu J (1974) Filariasis on Kinmen (Quemoy) Islands, Republic of China. II. Clinical investigations. Southeast Asian J Trop Med Public Health 5: 398-407.

22. Iyengar MO, de R, van DW (1959) Interruption of transmission of anopheles-borne filariasis by indoor residual spraying in Netherlands New Guinea. Trop Geogr Med 11: 287-290.

23. Self LS, Usman S, Sajioiman H, Partono F, Nelson MJ, et al. (1978) A multidisciplinary study on bancroftian filariasis in Jakarta. Trans R Soc Trop Med Hyg 72: 581-587.

24. Wolfe MS, Aslamkhan M (1972) Bancroftian filiariasis in two villages in Dinajpur District, East Pakistan. I. Infections in man. Am J Trop Med Hyg 21: 22-29.

25. Aslamkhan M, Wolfe MS (1972) Bancroftian filariasis in two villages in Dinajpur District, East Pakistan. II. Entomological investigations. Am J Trop Med Hyg 21: 30-37.

26. Enarson DA, Enarson PM (1982) Filariasis in an upland population in the Philippines. Trop Geogr Med 34: 353-358.

27. Harinasuta C, Sucharit S, Deesin T, Surathin K, Vutikes S (1970) Bancroftian filariasis in Thailand. A new endemic area. Southeast Asian J Trop Med Public Health 1: 233-245.

28. Barry C, Ahmed A, Khan AQ (1971) Endemic filariasis in Thakurgaon, East Pakistan. Am J Trop Med Hyg 20: 592-597.

29. Pani SP, Das LK, Balakrishnan N, Sadanandane C, Rajavel AR, et al. (1989) A study on the clinical manifestation of Bancroftian filariasis in Pondicherry, South India. Indian Med Gaz 123: 111-115.

30. Pani SP, Balakrishnan N, Srividya A, Bundy DA, Grenfell BT (1991) Clinical epidemiology of bancroftian filariasis: effect of age and gender. Trans R Soc Trop Med Hyg 85: 260-264.

31. Rao CK, Rao PK, Prasad KR (1980) Epidemiological studies on bancroftian filariasis in East Godavari district (Andhra Pradesh): II. Periodicity of microfilaria. J Commun Dis 12: 164-166.

32. Chand D, Singh MV, Gupta BB, Srivastava RN (1961) A note on filariasis in Gonda Town (Utter Pradesh). Indian J Malariol 15: 39-47.

33. Nair CP (1962) Filariasis in Kerala State. VII. Survey of Ponani (Palghat District). Indian J Malariol 16: 47-62.

34. Joseph C, Peethambaran P (1963) A Filariasis Survey of Trichur (Kerala State). Indian J Malariol 17: 33-36.

35. Chand D, Singh MV, Pathak VK (1961) Problem of filariasis in the District of Deoria (Uttar Pradesh). Indian J Malariol 15: 31-38.

36. Chand D, Singh MV, Pathak VK (1961) Filariasis in the District of Ghazipur (Uttar Pradesh). Indian J Malariol 15: 21-29.

37. Varma BK, Sinha VP, Dass NL (1960) Filariasis in Sultanganj and its suburbs (Bihar). Bull Nat Soc Mal Mosq Dis 8: 149-152.

38. Chand D, Singh MV, Vyas LC (1962) Filariasis in Gorakhpur District, Uttar Pradesh. Indian J Malariol 16: 269-276.

39. Raghavan NG (1951) Filariasis in Porbandar, Saurashtra. Indian J Malariol 5: 203-207.

40. Chand D, Singh MV, Shrivastava RN (1961) Filariasis in Bahraich District, Uttar Pradesh. Indian J Malariol 15: 175-184.

41. Sarma RV, Vallishayee RS, Mayurnath S, Narayanan PR, Radhamani MP, et al. (1987) Prevalence survey of filariasis in two villages in Chingleput district of Tamil Nadu. Indian J Med Res 85: 522-530.

42. Ramaiah KD, Pani SP, Balakrishnan N, Sadanandane C, Das LK, et al. (1989) Prevalence of bancroftian filariasis & its control by single course of diethyl carbamazine in a rural area in Tamil Nadu. Indian J Med Res 89: 184-191.

43. Nair CP (1961) Filariasis in centrally administered areas. II. Survey of Laccadive, Minicoy and Aminidivi Islands. Indian J Malariol 15: 263-283.

44. Krishnaswami AK (1955) Filariasis in Mangalore (South India). Indian J Malariol 9: 1-16.

45. Rath RN, Das RK, Mishra G, Mohapatra BN, Ramakrishna C (1984) Bancroftian filariasis in two selected rural communities in Puri district: Orissa--a comparative study of filariometric data. J Commun Dis 16: 104-112.

46. Nair CP, Radhagovinda R, Joseph C (1960) Filariasis in Kerala State. VI. Filaria survey of Fort Cochin municipality. Indian J Malariol 14: 223-231.

47. Russel S, Das M, Rao CK (1975) Filariasis in Andaman and Nicobar islands. Part I - Survey findings - Nancowry, Terressa, Chowra, Carnicobar and Port Blair. J Commun Dis 7: 15-30.

48. Singh MV, Rastogi KC, Singh RP, Srivastava VK (1963) Observations on Rural Filariasis in Sitapur District (Uttar Pradesh). Indian J Malariol 17: 303-310.

49. Gubler DJ, Bhattacharya NC (1974) A quantitative approach to the study of Bancroftian filariasis. Am J Trop Med Hyg 23: 1027-1036.

50. Dondero TJ, Jr., Bhattacharya NC, Black HR, Chowdhury AB, Gubler DJ, et al. (1976) Clinical manifestations of Bancroftian filariasis in a suburb of Calcutta, India. Am J Trop Med Hyg 25: 64-73.

51. Rozeboom LE, Bhattacharya NC, Gilotra SK (1968) Observations on the transmission of filariasis in urban Calcutta. Am J Epidemiol 87: 616-631.
