# Supplementary material for: Global Eradication of Lymphatic Filariasis: The Value of Chronic Disease Control in Parasite Elimination Programmes
Source: PLoS One. 2008 Aug 13;3(8):e2936. doi: 10.1371/journal.pone.0002936 (PMC2490717; doi:10.1371/journal.pone.0002936)
Supplement: Text S1 — Adequacy of fit of the logistic dose-response regression model with a threshold (0.03 MB DOC) [file pone.0002936.s002.doc]

We used the Hosmer and Lemeshow test [1] for assessing the goodness of fit of the threshold model specified in the text. This test is based on grouping the data into percentile groups (eg., deciles) of *X* (mf prevalence (%) here), estimating both the mean proportion of diseased response predicted by the fitted model and the mean fraction of disease observed for individuals in each group, and performing an ordinary χ^2^ test for comparing the predicted numbers diseased against the observed number of individuals diseased using *g* – (*p* + 1) d.f., where *g* is the percentile groups used and *p* denotes the number of parameters in the fitted model. The predicted (curve) versus variously estimated observed proportions of disease (on a logit scale) in relation to mf prevalence (%) values are plotted in Figure A1, and graphically shows that, apart from a slight tendency to overestimate probabilities at low mf prevalences and heterogeneous data points between mf prevalences 25 –40%, the present logistic regression model with a threshold fits the data sufficiently well whether based on 1) individual observed logit diseased proportions from each study (open circles) and 2) mean logit proportions based on grouping *X* into pentiles (closed circles). The values of the χ^2^ statistic and *p* value shown on the graph were obtained by applying the χ^2^ test described above for the pentile data (division into 5 groups required to increase sample size within each group to ~ 10-20 subjects per group [2]), and support the visual impression from the figure that the present model provides an adequate fit to the observed data. A test applied to grouping the data into deciles also provided a good model fit to the observed data (χ^2^ = 0.347, *p* = 0.556).

REFERENCES

1. Hosmer DW, Lemeshow S (1980) Goodness-of-fit tests for the multiple logistic regression model. Comm Stat - Theor Meth 9: 1043-1069.

2. Harrell FE (2001) Regression Modeling Strategies. New York: Springer-Verlag.
