# Supplementary figures and images for: Global Eradication of Lymphatic Filariasis: The Value of Chronic Disease Control in Parasite Elimination Programmes
Source: PLoS One. 2008 Aug 13;3(8):e2936. doi: 10.1371/journal.pone.0002936 (PMC2490717; doi:10.1371/journal.pone.0002936)

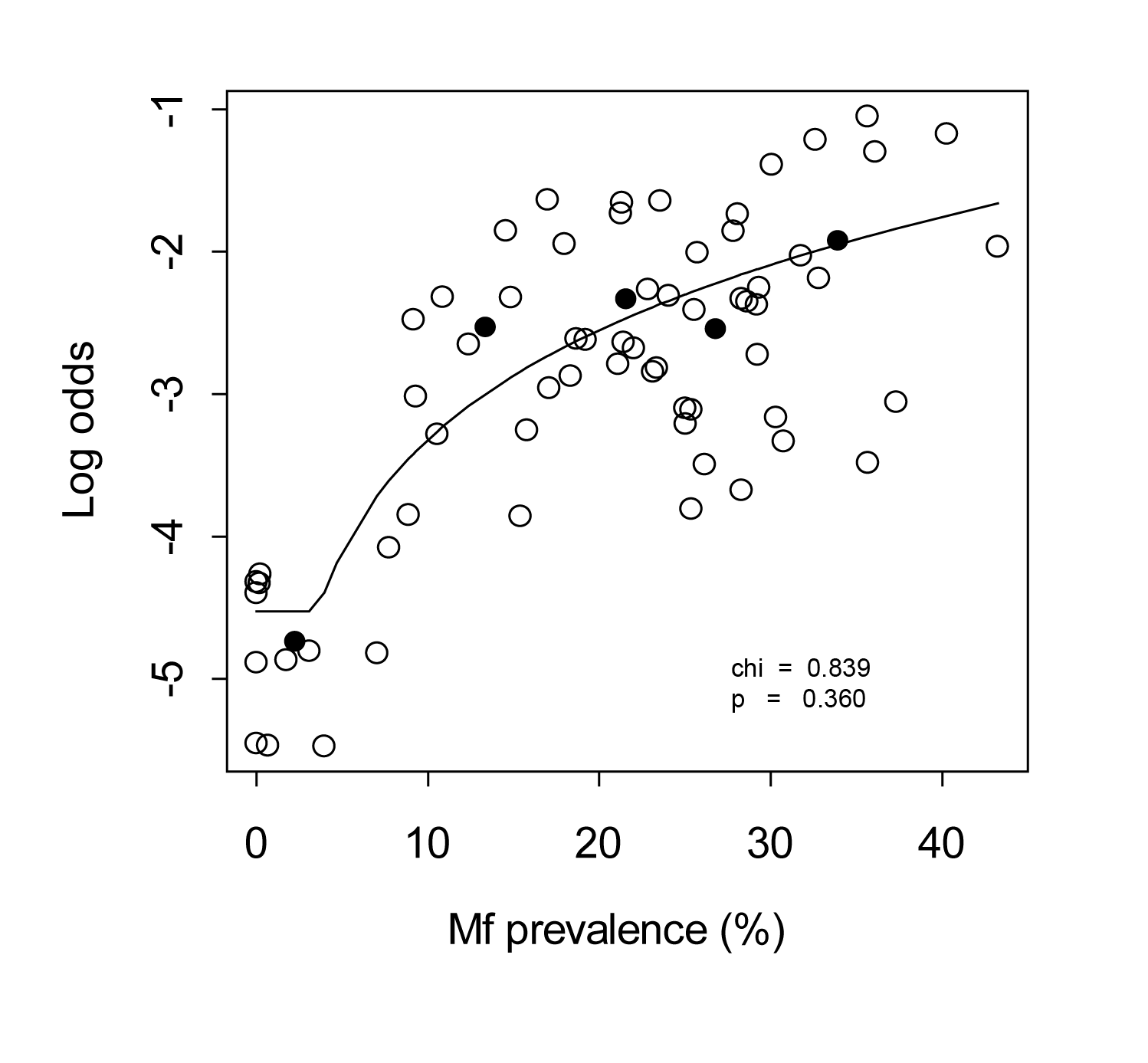

Supplement: Figure S1 — Logit proportions ( = log odds) of chronic LF disease against individual study (open circles) and pentiles (closed circles) of mf prevalence (%) values. The figures shown on the graph represent the chi-square statistic and p - values obtained by applying the chi-square test described in the text for data grouped into pentiles of mf prevalence. (6.73 MB DOC) [file pone.0002936.s003.doc]
